# Immunological markers in type 1 diabetes mellitus in Thi-Qar province, southern Iraq

**DOI:** 10.25122/jml-2021-0387

**Published:** 2022-10

**Authors:** Ghaneemah Malik Hamadi

**Affiliations:** 1 Department of Community Health, Nasiriyah Technical Institute, Southern Technical University, Nasiriyah, Iraq

**Keywords:** type1 diabetes, biomarkers, β-cell

## Abstract

Type 1 diabetes mellitus (T1D) is a chronic autoimmune illness defined as insulin insufficiency resulting from the autoimmune breakdown of pancreatic beta cells producing insulin in the islets of Langerhans. Biomarkers are markers of physiological or pathological processes that are normal or abnormal, playing a crucial function in clinical evaluation, prognosis, and therapy response monitoring. This study aimed to investigate some biomarkers associated with T1D and examine the association between glutamic acid carboxylase (GADA) antibody and islet antigen-2 autoantibody (IA-2A) for β-cell stress and death in patients with T1D. The current study included 60 patients with T1D, 32 (53.33%) males and 28 (46.67%) females between 9 to 18 years old, and 30 healthy individuals as control. Glutamic acid carboxylase, islet antigen-2 autoantibody and connecting peptide levels in the blood were evaluated. Positive results for IA-2A and GADA were shown in 89.04% and 38% of T1D patients, respectively. The normal level frequency and C-peptide titer mean were significantly lower between T1D and healthy control. However, no statistically significant changes were observed in the C-peptide level among GADA positive and negative patients. Finally, the C-peptide concentrations were significantly lower for positive IA-2A compared to negative IA-2A persons. The combination of IA-2A, GADA, and C-peptide could indicate stronger diagnostic measures at a low cost for patients with T1D.

## INTRODUCTION

Type 1 diabetes (T1D) is classically known as insulin insufficiency caused by autoimmune damage to pancreatic beta cells [1]. Recent research has hypothesized that beta cells are vigorously involved in causing illness, indicating the need to improve classical models of T1D pathogenesis [2–3]. Unfortunately, the majority of β-cells are lost in phases of preclinical T1D and appear too late after diagnosis when approximately 90% of beta cells have been destroyed. To stop beta cells from being destroyed, specific immunotherapy has been used for minimal preservation of beta cell activity without actual recovery from T1D [4]. T1D results from various factors, including the environment, genetics, triggers, and modifying factors.

On the other hand, none of these factors can be used as a definitive diagnostic standard. The exiting biomarkers for T1D diagnosis still depend on hyperglycemia effects, like hemoglobin from elevated glucose, and other biomarkers that differentiate T1D from other subgroups of diabetes, like low levels of C-peptide or autoantibodies. Only 5–10% of diabetes types and subtypes account for T1D. Usually, these can be distinguished from type 2 diabetes mellitus ((T2D) and another subtype through specific autoantibodies. There are several autoantibody markers that are routinely employed in the diagnosis of T1D [5, 6], which include glutamic acid decarboxylase autoantibody (GADA), insulinemia 2 associated autoantibody (IA-2A) and others. At least one autoantibody exists in more than 95% of persons with type 1 proteinuria when hyperglycemia is detected [7]. Autoantibodies are used for more than just diagnosis and markers for illness classification, and they are also considered the gold standard for predicting the progression of T1D. Autoantibody phenotype and numbers, epitope, familiarity, and order of appearance, along with genotypes of patients and their ages, all take part in T1D risk [8]. IA-2A and GADA, for example, are the most commonly discovered autoantibodies for children; however, they vanish in twenty-five percent and respectively ten percent of children cases at the time of clinical start. Whereas just a minor percentage of children showed IA-2A, but ZnT8A antibodies as the initial autoantibody, each proceeded with the diagnosis [9–11]. The level of serum C-peptide level (which acts as a substitute for insulin) is a reliable and sensitive indicator for beta-cell activity; it can also be used to distinguish self-immune diabetes mellitus from the other subtypes of DM (diabetes mellitus) [12]. Developing accurate blood biomarkers, particularly those that reflect pancreatic beta cell death or stress, is challenging since T1D, caused by an autoimmune attack of beta cells, accounts for just around 0.002 percent of body mass [13]. Although T1D has been studied for over a century, the origin of the illness is still unknown. To date, HbA1c, glucose, connecting peptides, and autoantibodies have been primarily indicators of the disease in clinical practice, although autoantibodies act as comparatively good predictive markers of eventual disease. At the same time, there was progress in understanding the etiology of T1D [14]. This study investigated some biomarkers associated with T1D and the relationship between specific glutamic acid carboxylase (GADA) and islet antigen-2 autoantibody (IA-2A) with beta cell stress and/or death among T1D patients.

## Material and Methods

### Participants

The sample included 60 patients, 32 (53.33%) males and 28 (46.67%) females with ages between 9 to 18 years old, who were attending the Specialized Diabetes and Endocrinology Center in Thi-Qar Province, southern Iraq, from April 2021 to October 2021, and 30 healthy individuals as a control.

### Methods

A total of 5 ml of blood was drawn via vein puncture from each participant. Collected samples were allowed to coagulate at room temperature, and the serum was then centrifuged for 10 minutes at a rate of 1500 rounds per minute to yield serum. Each sample was separated into numerous aliquots and kept at -20℃ until the serological examination was required.

### Measurement glutamic acid carboxylate antibody (GADA)

Anti-GAD 65 autoantibody ELISA Kit was designed to determine the amount of antibodies in the serum. The detection and calibration of GAD auto-Abs were performed according to the manufacturer's protocol (GAD auto-Abs, Demeditec, Germany). Anti-glutamic acid decarboxylase antibodies in the serum of patients, calibrators and controls were allowed to react with the glutamic acid decarboxylase 65-coated antigen in the wells of the ELISA plate in the anti-GAD ELISA kit. Samples were discarded after 1 hour of incubation, leaving anti-GAD antibodies conjugated to plate-fixed GAD65 antigen. In the second incubation stage, GAD65- biotin antigen was added, and a bridge was formed between the fixed GAD65-antigen plate and GAD65-biotin via the capacity of the anti-GAD antibody in the samples to behave differently. In a further incubation stage, the quantity of GAD65-biotin antigen bound was evaluated by adding streptavidin peroxidase, which bound selectively to biotin. Unbounded peroxidase and excess were washed away, and TMB (tetramethylbenzidine) was added to produce a blue color. The addition of the halt solution, which causes the contents of the well to become yellow, stopped this reaction. Then an ELISA plate reader was used to measure the absorbance of the yellow reaction mixture at 450 nm (nanometers). The presence of anti-GAD antibody in the test samples was indicated by greater absorption. 5–2000 U (units)/ml was the measurement range.

### Measurement islet antigen-2 autoantibody (IA-2A)

The detection of IA-2A auto-Abs was done according to the manufacturer's protocol (Human, Cusabio, China Kit) using qualitative indirect immunoassay technology. This kit includes an antigen-pre-coated microtiter plate. The anti-human immunoglobulin conjugated HRP (Horseradish Peroxidase) was pumped into the wells. Any antibodies particular to the current antigen would bind to the previously covered antigen. After removing any unbound reagent by washing, a substrate solution was added to the wells, and color was established according to the quantity of human IA-2A bound in the first stage. When color conversion halted, color intensity was measured.

### C-peptide measurement

Detection and titration of C-peptide serum were done according to the manufacturer's protocol (C-peptide, Demeditec, Germany). The kit of C-peptide is a solid-phase enzyme immunosorbent assay that works on the concept of competitive binding. Anti-mouse antibodies were encapsulated in microtiter wells, binding monoclonal antibodies targeting an exact antigen location on the C-peptide molecule. The patient's endogenous C-peptide was completed with the C-peptide-horseradish peroxidase conjugate to bind to the coated antibody. The unbound conjugate was rinsed away after incubation. The C-peptide concentration in the sample has an opposite relationship with the amount of peroxidase-bound conjugate. The intensity of the color formed after adding the substrate solution was inversely proportional to the C-peptide concentration in the sample of the patient.

### Statistical analysis

The statistical analysis was carried out using SPSS version 24. Descriptive statistics such as frequencies, relative frequencies, means and standard deviations were involved. The statistical Chi-Square test, simple correlation (r), and simple linear regression were used to evaluate the associations between parameters. Results were statistically significant at the probability level of P≤0.05.

## Results

### Autoantibodies

Anti-glutamic acid decarboxylase antibody results are shown in Table 1. The positive rate of anti-GAD antibodies among patients with T1D (89.04%) was significantly higher than in healthy controls (HC) (4.7%) (P<0.05). There was a significantly elevated mean titer (P<0.05) (687.9 U/ml) for anti-GAD antibodies in the T1D group compared to the HC (1.9 U/ml).

**Table 1:** Mean titer and frequency of anti-glutamic acid decarboxylase (AGD).

Parameters	AGD Abs			P-value
	Positive	Negative	Mean	
**T1D**	89.04%	10.96%	687.9	P<0.05
**HC**	4.7%	95.3%	1.9	

The positive rate of IA-2A was significantly higher in persons with T1D (38%) compared to the HC group (4.7%) (P<0.05) (Table 2).

**Table 2:** Frequency of islet antigen-2 autoantibody (IA-2A).

Parameters	IA-2A		P-value
	Positive	Negative	
**T1D**	38%	62%	P<0.05
**HC**	4.7%	95.3%	

### Connecting peptide

Most patients with T1D had significantly lower than normal C. peptide serum levels (67.15%) compared with HC (4%) (P<0.05). Regarding the mean C. peptide titer, as shown in Table 3, the lowest level was in patients with T1D (3.3 ng/ml), followed by HC (7.2 ng/ml) with statistical differences (P<0.05).

**Table 3:** Frequency and mean titer of connecting peptide (C-P).

Parameters	Connecting Peptide (C-P)				P-value
	Lower than normal (<3)	Normal (3–6)	Higher than normal (>6)	Mean titer	
	Frequency %	Frequency %	Frequency %		
**T1D**	67.15	30.85	2	3.3	P<0.05
HC	4	32	64	7.2	

### Association of C-peptide with other biomarkers

The relationship between anti-GAD Abs and serum C-peptide between persons with T1D and healthy persons is shown in Table 4. There was no significant relationship (P>0.05) between patients with anti-GAD positive (low, medium, high) or anti-GAD Abs negative with frequency ratio for the level of C-peptide between T1D and HC individuals. For the C-peptide medium titer, the same results were reported.

**Table 4:** Association among anti-glutamic acid decarboxylase autoantibody (anti-GAD Ab) and connecting peptide (C-P).

				Connecting peptide (C-P)								P-value
				L. than N(<3)		Normal (3–6)		H. than N(>6)		Total		
				Freq. (%)	Mean titer	Freq. (%)	Mean titer	Freq. (%)	Mean titer	Freq. (%)	Mean titer	
**Anti-GAD Ab (U/ml)**	T1D (nu.=60)	+ve	L (nu.=11)	6 (54.5)	1.4	5 (45.5)	4.3	0 (0)	0	11 (100)	3.3	P>0.05
			M (nu.=7)	6 (85.7)	1.5	1 (14.3)	6.6	0 (0)	0	7 (100)	3.3	
			H (nu.=33)	21 (63.6)	1.7	10 (30.3)	4.2	2 (6.1)	8.1	33 (100)	3.4	
			T (nu.=51)	33 (64.7)	1.6	16 (31.4)	5.1	2 (3.9)	8.1	51 (100)	3.4	
		-ve (nu.=9)		8 (88.9)	3.1	1 (11.11)	4.2	0 (0)	0	9 (100)	3.2	
		Total (nu.=60)		41 (68.3)	2.8	17 (28.3)	4.9	2 (3.3)	8.1	60 (100)	3.4	
	HC (nu.=30)	Positive	L (nu.=1)	0 (0%)	0	1 (100)	4.1	0 (0%)	0	1 (100)	4.1	P>0.05
			M (nu.=0)	0 (0%)	0	0 (0%)	0	0 (0%)	0	0 (0%)	0	
			H (nu.=0)	0 (0%)	0	0 (0%)	0	0 (0%)	0	0 (0%)	0	
			T (nu.=1)	0 (0%)	0	1 (100)	4.1	0 (0%)	0	1 (100)	4.1	
		Negative (nu.=29)		1 (3.4)	3.2	11 (37.9)	6.4	17 (58.6)	8.3	29 (100)	7.4	
		Total (nu.=30)		1 (3.3)	3.2	12 (40)	6.2	17 (56)	8.3	30 (100)	7.3	

L – Low; M – Moderate; H – High; T – Total; N – Normal; Freq – Frequency; nu – number.

**Figure 1:**
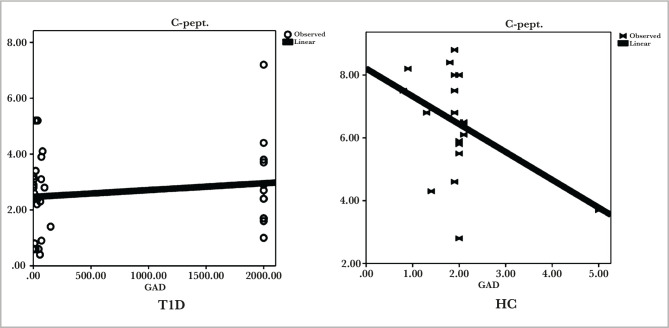
Regression analysis of anti-glutamic acid decarboxylase antibody and connecting peptide in patients with T1D and healthy controls.

In the T1D group, the regression analysis in Figure 1 showed a slight positive correlation between the level of anti-GAD antibodies and the level of C-peptide with no significant differences (p>0.05). Conversely, for the HC group, there was a negative correlation between the antibodies level of anti-GAD and the level of C-peptide with no significant differences (p>0.05).

The association between IA-2A and connecting peptides in patients and controls is displayed in Table 5. IA-2A positive for patients with T1D and healthy control was 20/23 (86.9%) and 0/1 (0%), respectively. Lower than normal levels of connecting peptides and total mean titer were 3.1 ng/ml in T1D and 5.3 ng/ml in HC. 54.1% (20/37) were IA-2A negative in the T1D group and 3.45% (1/29) in the HC group. Lower than normal levels of connecting peptides and total mean titer were 4 ng/ml in T1D and 7.6 ng/ml in HC. Differences in the level of C-peptide between IA-2A positive and IA-2A negative were significant (P<0.05).

**Table 5:** Correlation between islet antigen-2 autoantibody (IA-2A) and connecting peptide (C-P).

Parameters			Connecting Peptide (C-P) (ng/ml)								P-value
			L. than N (>3)		Normal (3–6)		H. than N (>6)		Total		
			Freq. (%)	Mean	Freq. (%)	Mean	Freq. (%)	Mean	Freq. (%)	Mean	
**IA-2A**	T1D (n=60)	Positive (n=23)	20 (86.9)	2.6	3 (13)	4.4	0 (0)	0	23 (100)	3.1	P<0.05
		Negative (n=37)	20 (54.1)	2.6	16 (43.2)	4.7	1 (2.7)	8.1	37 (100)	4.1	
		Total (n=60)	41 (68.3)	2.6	18 (30)	4.7	1 (1.7)	8.1	60 (100)	3.7	
	HC (n=30)	Positive (n=1)	0 (0)	0	1 (100)	5.1	0 (0)	0	1 (100)	5.3	P<0.05
		Negative (n=29)	1 (3.4)	3.6	10 (34.5)	6.1	18 (62.1)	8.5	29 (100)	7.6	
		Total (n=30)	1 (3.3)	3.6	11 (36.7)	6	18 (60)	8.5	30 (100)	7.5	

N – Normal; Freq – Frequency; L – Low; H – High.

## Discussion

T1D is an autoimmune disease marked by the loss of beta cells due to autoimmune processes, as evidenced by the onset of the autoimmune disease of the pancreas. Eliminating these cells results in an irreversible loss of endogenous insulin production, necessitating daily administration of exogenous insulin [15]. One theory for T1D is that pancreatic beta cells are destroyed due to a defect in immune control resulting from infection or environmental factors that stimulate the immune system of people with a genetic predisposition to form an autoimmune reply against the altered pancreatic beta cells [16]. Both humoral and cellular islet autoimmunity is linked with type 1 diabetes, and poor immunological control appears to play a role [17].

Type 1 diabetes is often linked to anti-GAD, islet cell or insulin abscess, indicative of autoimmune processes that result in beta cell loss [15]. Glutamic acid decarboxylase is a biosynthetic enzyme for the inhibitory neurotransmitter gamma-aminobutyric acid (GABA) and has been well-studied [18]. The GAD65 isoforms are expressed only in human pancreatic islet cells. Glutamic acid decarboxylase converts glutamic acid to GABA enzymatically in GABAergic neurons and islet cells. As a result, the autoantibodies against the GAD65 isoform are used to evaluate human disease [19].

The current research showed that the highest recurrences of positive anti-GAD antibodies and IA-2A were 89.04% and 38% among T1D patients, respectively. The study by Kawasaki et al. [20] supports these findings, showing significantly higher GAD antibodies and IA-2A antibodies among patients with T1D than in the HC group. This result contradicts the findings of Mehdi et al. [16] for the same biomarkers. Another study [21] conducted in Saudi Arabia reported a positive recurrence rate for IA-2A of 27%, and GADA had a positive recurrence rate of 54%. A research in Taiwan found that GADA was 47% and IA-2A was 23% among T1D patients [22]. The occurrence of autoantibodies to glutamic acid carboxylase and IA-2A in patients with T1D was 72.2% and 41.1 respectively, in the study by Kawasaki et al. [23]. These differences in the percentage of antibody frequency in many studies may be attributable to various factors related to the patient's genetic constitution, the environment, and the sensitivity of the test. The discovery of autoantibodies against beta-cell antigens indicates the onset of autoimmunity targeting insulin-producing cells [24].

The use of C-peptide as a direct indicator of the cell damage caused by autoimmune processes has originated [25], and it is generally known that it plays a crucial role in insulin production [26]. The levels of connecting peptides are good markers of blood insulin levels and pancreatic cell activity [27]. A lower concentration of C-peptide was reported among T1D patients compared to the HC group in this study, consistent with Tang [28] and Lebastchi and Herold [25]. Only a few prior investigations have shown that C-peptide levels gradually decline over time as the disease progresses, eventually reaching an immeasurable level years after the onset of the disease. Several studies [29, 30] indicated that C-peptide levels slowly decreased during pre-diabetes and subsequently increased throughout the clinical stages. Variation in C-peptide concentrations between children might be more complex that may be explained by the age-related occurrence of C-peptide rise, which is construed as no rise in child growth is equal to a decrease in this biomarker [31]. Many studies have shown that autoantibodies, primarily GADA and IA-2A, have an effect on cell survival and remaining function (exemplified by the low C-peptide level in this research). Several investigators found contradictory results regarding autoantibody levels, residual mass, and function of cells, revealing no association, negative correlation or positive correlation [32–34].

The current investigation found no significant variations in the concentration of C-peptide between study groups, whether GADA positive or negative. These contradictory results may be due to the difference in sample inclusion criteria (*e.g*., the patient's age at the time of diagnosis) and biomarker assessment methods. The results of Table 5 completely agree with Christie et al. [35], who reported that positive islet antigen-2 autoantibody might be associated with a pronounced decrease in the mass and residual activity of β-cells. There are several possible justifications for lower connecting peptide levels in IA-2A positive compared with IA-2A negative people. First, IA-2A significantly impacts insulin secretion as IA-2A deficiency and/or IA-2A deficiency results in decreased insulin level and secretion due to a reduction in the number of dense core vesicles [36]. Additionally, IA-2A, which is frequently linked with other antibodies, increases the potential risk for rapid progression to clinical manifestation compared to the presence of numerous antibodies. It is suggested that IA-2A formation is linked to a captive shift in T1D progression, in which only the intracellular portion of IA-2A may encounter the immune system on the cell's outer surface if there is cell damage or dysfunction [37].

Finally, in T1D patients, the glutamic acid carboxylase antibody and islet antigen-2 autoantibody and connecting peptide combination can be promoted as the most reliable diagnostic method that is inexpensive. The existence of islet antigen-2 autoantibody in patients' blood could reveal cell stress and/or death through the initiating and initial stages of T1D as islet antigen-2 autoantibody-positive was inversely related to cell vital role (as exemplified by the level of C-peptide). In addition, glutamic acid carboxylase antibodies did not influence connecting peptide levels in the research groups.

## Personal thanks

I thank the patients and their families for cooperating during the study period. I also thank the Specialized Center for Diabetes and Endocrinology staff in Thi-Qar Province in southern Iraq for their support during the sample collection.

## References

[1] DiMeglio LA, Evans-Molina C, Oram RA (2018). Type 1 diabetes. Lancet.

[2] Atkinson MA, Bluestone JA, Eisenbarth GS, Hebrok M (2011). How does type 1 diabetes develop? The notion of homicide or beta-cell suicide revisited. Diabetes.

[3] Soleimanpour SA, Stoffers DA (2013). The pancreatic beta cell and type 1 diabetes: innocent bystander or active participant?. Trends Endocrinol Metab.

[4] Raghavendra GM, Emily KS, Farooq S, Carmella E (2016). Biomarkers of β-Cell Stress and Death in Type 1 Diabetes. Curr Diab Rep.

[5] Schlosser M, Mueller PW, Achenbach P, Lampasona V (2011). Diabetes Antibody Standardization Program: First evaluation of assays for autoantibodies to IA-2beta. Diabetes Care.

[6] Lampasona V, Schlosser M, Mueller PW, Williams AJ (2011). diabetes antibody standardization program: first proficiency evaluation of assays for autoantibodies to zinc transporter 8. Clin Chem.

[7] Ziegler AG, Rewers M, Simell O, Simell T (2013). Seroconversion to Multiple Islet Autoantibodies and Risk of Progression to Diabetes in Children. Jama-J Am Med Assoc.

[8] Bonifacio E (2015). Predicting type 1 diabetes using biomarkers. Diabetes Care.

[9] Ilonen J, Lempainen J, Hammais A, Laine AP (2018). Primary islet autoantibody at initial Seroconversion and autoantibodies at diagnosis of type 1 diabetes as markers of disease heterogeneity. Pediatr Diabetes.

[10] Endesfelder D, Hagen M, Winkler C, Haupt F (2016). A novel approach for the analysis of longitudinal profiles reveals delayed progression to type 1diabetes in a subgroup of multiple islet-autoantibody-positive children. Diabetologia.

[11] Vehik K, Lynch KF, Schatz DA, Akolkar B (2016). Reversion of beta-Cell Autoimmunity Changes Risk of Type 1 Diabetes: TEDDY Study. Diabetes Care.

[12] Leighton E, Sainsbury CAR, Jones GC (2017). A Practical Review of C-Peptide Testing in Diabetes. Diabetes Ther.

[13] Saisho Y, Butler AE, Manesso E, Elashoff D (2013). Beta-cell mass and turnover in humans: effects of obesity and aging. Diabetes Care.

[14] Atkinson MA, von Herrath M, Powers AC, Clare-Salzler M (2015). Current concepts on the pathogenesis of type 1 diabetes--considerations for attempts to prevent and reverse the disease. Diabetes Care.

[15] Habtamu WB (2015). Classification, pathophysiology, diagnosis and management of diabetes mellitus. Journal of Diabetes and Metabolism.

[16] Mahdi NK, Al-Abadi HL, Al-Naama LM, Mahdi JK, Alawy M (2015). The Role of Autoantibody and Antioxidant Enzymes in Patients with Type I Diabetes. Medical Journal of Islamic World Academy of Sciences.

[17] Mathis D, Benoist C (2004). Back to central tolerance. Immunity.

[18] Yoon J, Jun H (2005). Autoimmune destruction of pancreatic β cells. American Journal of Therapeutics.

[19] Skoglund C (2011). Autoantibodies related to type 1diabetes in children (thesis).

[20] Kawasaki E, Oikawa Y, Okada A, Kanatsuna N (2020). Zinc transporter 8 autoantibodies complement glutamic acid decarboxylase and insulinom associated antigen-2 auto antibodies in the identification and characterization of Japanese type 1 diabetes. J Diabetes Investig.

[21] Damanhouri LH, Dromey JA, Christie MR, Nasrat HA (2005). Autoantibodies to GAD and IA-2 in Saudi Arabian patients with diabetes. Diabet Med.

[22] Chang YH, Shiau MY, Tsai ST, Lan MS (2004). Autoantibodies against IA-2A, GAD and topoisomerase II in type 1 patients with diabetes. Biochemical and Biophysical research communications.

[23] Kawasaki E, Oikawa Y, Okada A, Kanatsuna N (2021). Different interaction of onset age and duration of type 1 diabetes on the dynamics of autoantibodies to insulinoma-associated antigen-2 and zinc transporter 8. J Diabetes Investig.

[24] Couper JJ, Haller MJ, Ziegler AG, Knip M (2014). ISPAD Clinical Practice Consensus Guidelines 2014 Compendium. Phases of type 1 diabetes in children and adolescents. Pediatr Diabetes.

[25] Lebastchi J, Herold CK (2012). Immunologic and Metabolic Biomarkers of β-Cell Destruction in the Diagnosis of Type 1 Diabetes. Cold Spring Harb. Perspect. Med.

[26] Wahren J, Kallas Å Sima AFA (2012). The clinical potential of c-peptide replacement in type 1 diabetes. Diabetes.

[27] Shetty V, Jain HR, Singh G, Parekh S, Shetty S (2017). C-peptide levels in diagnosis of Diabetes mellitus: A case-control study. International Journal of Scientific Study.

[28] Tang W, Liang H, Cheng Y, Yuan JO (2021). Diagnostic value of combined islet antigen reactive T cells and autoantibodies assays for type 1 diabetes mellitus. J Diabetes Investig.

[29] Tsai EB, Sherry NA, Palmer JP, Herold KC (2006). The rise and fall of insulin secretion in type 1 diabetes mellitus. Diabetologia.

[30] Sosenko JM, Palmer JP, Rafkin-Mervis L, Krischer JP (2008). Glucose and C-peptide changes in the perionset period of type 1 diabetes in the Diabetes Prevention Trial-Type 1. Diabetes Care.

[31] Sosenko JM, Palmer JP, Greenbaum CJ, Mahon J (2006). Patterns of metabolic progression to type 1 diabetes in the Diabetes Prevention Trial-Type 1. Diabetes Care.

[32] Sabbah E, Savola K, Kulmala P, Veijola R (1999). Diabetes-associated auto antibodies in relation to clinical characteristics and natural course in children with newly diagnosed type 1 diabetes. The Childhood Diabetes in Finland Study Group. J Clin Endocrinol Metab.

[33] Decochez K, Keymeulen B, Somers G, Dorchy H (2000). Use of an Islet Cell Antibody Assay to Identify Type 1 Diabetic Patients with Rapid Decrease in C-Peptide Levels after Clinical Onset. Belgian Diabetes Registry. Diabetes Care.

[34] Ten KQ, Aanstoot HJ, Birnie E, Veeze H, Mul D (2016). GADA Persistence and Diabetes Classification. Lancet Diabetes Endocrinol.

[35] Christie MR, Mølvig J, Hawkes CJ, Carstensen B (2002). IA-2 Antibody-Negative Status Predicts Remission and Recovery of C-Peptide Levels in Type 1 Diabetic Patient Treated With Ciclosporin. Diabetes Care.

[36] Cai T, Hirai H, Zhang G, Zhang M (2011). Deletion of IA-2 and/or IA-2β Decreases Insulin Secretion by Reducing the Number of Dense Core Vesicles. Diabetologia.

[37] Herawati E, Susanto A, Sihombing CN (2017). Autoantibodies in Diabetes Mellitus. Molecular and Cellular Biomedical Sciences.

